# Supplementary material for: Establishment of a morphological atlas of the Caenorhabditis elegans embryo using deep-learning-based 4D segmentation
Source: Nat Commun. 2020 Dec 7;11:6254. doi: 10.1038/s41467-020-19863-x (PMC7721714; doi:10.1038/s41467-020-19863-x)
Supplement: Supplementary file 1 — Supplementary Information [file 41467_2020_19863_MOESM1_ESM.pdf]

Supplementary Information

Establishment of a morphological atlas of the *Caenorhabditis elegans* embryo using deep-learning-based 4D segmentation

Cao et al.

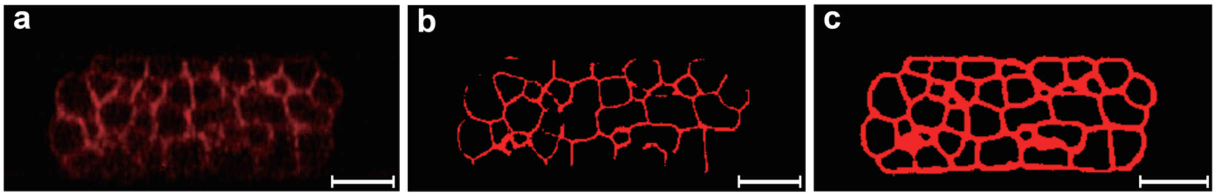

Supplementary Figure 1. Segmentation examples of B-CShaper and CShaper. Single-section views (left-right, anterior-posterior) of (**a**) a raw image of the mCherry membrane label, (**b**, **c**) the outputs from B-CShaper and CShaper, respectively. Scale bar, 10 μm.

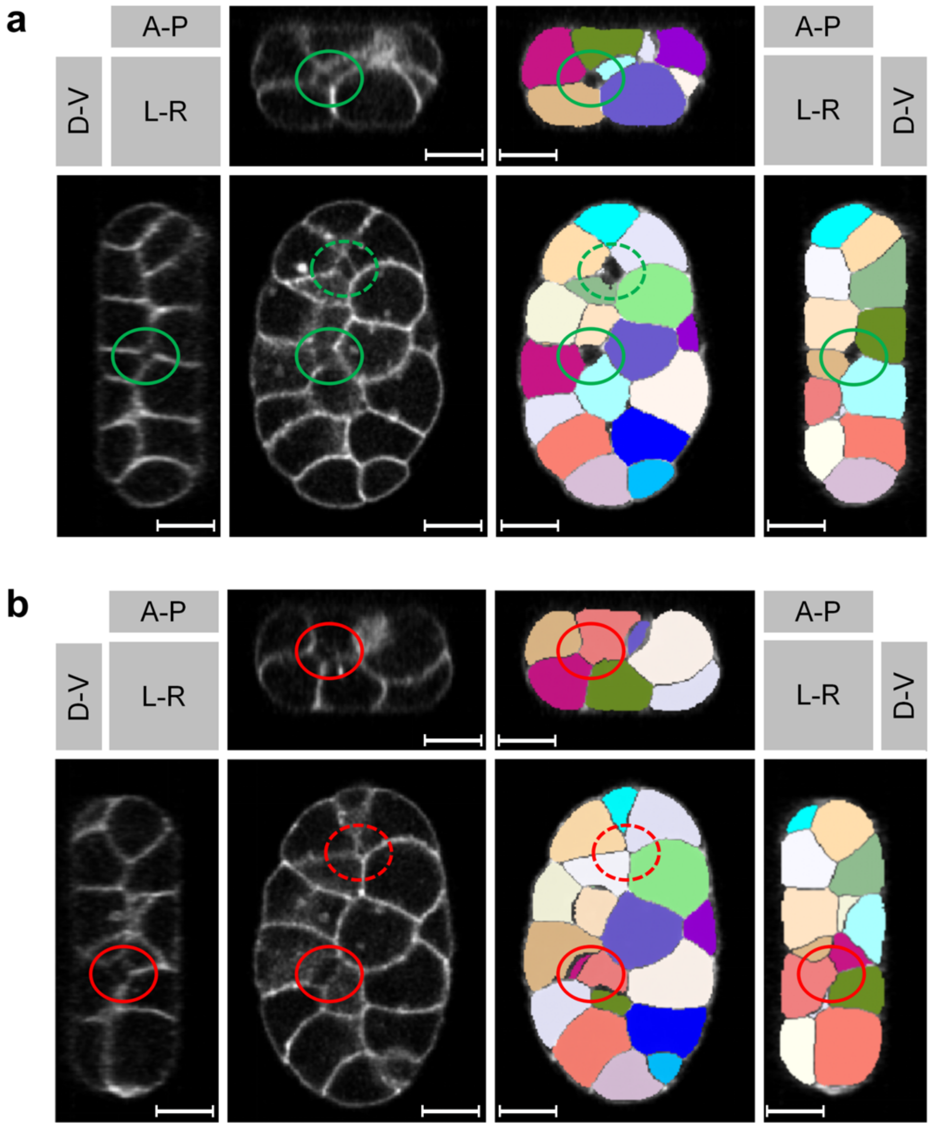

Supplementary Figure 2. The cavities within *C. elegans* embryos. Two cavities are reproducibly identified in (**a**) embryo Sample 08 at time point 32 and (**b**) embryo Sample 18 at time point 34 (Supplementary Data 1). The first cavity identified is located anteriorly in the embryo and is indicated by the dashed circles. The second cavity is located in the center of embryo and is indicated by the solid circles. Both cavities are segmented successfully in embryo Sample 08 but not in embryo Sample 18. In the segmented images, each color represents one specific blast cell. The view directions of both the fluorescent and segmented images are illustrated on the top left and top right, respectively. D, dorsal; V, ventral; L, left; R, right; A, anterior; P, posterior. Scale bar, 10 µm.

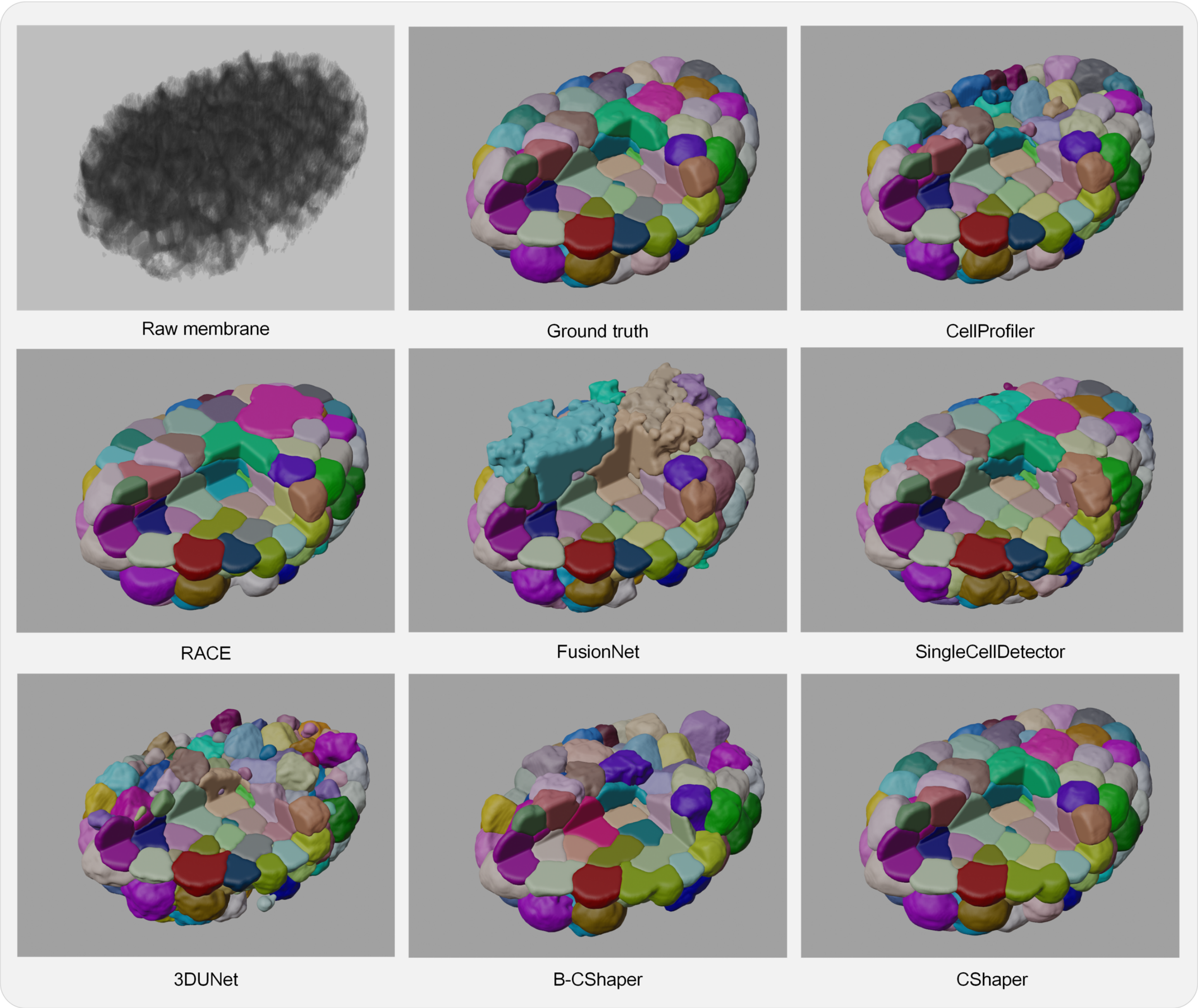

Supplementary Figure 3. Segmentation examples of different methods. *C. elegans* membrane stack is segmented by CellProfiler[1], RACE[2], FusionNet[3], SingleCellDetector[4], 3DUnet[5], B-CShaper and CShaper, respectively. For comparison, manual annotation is also listed as the ground truth. Each color represents one specific blast cell.

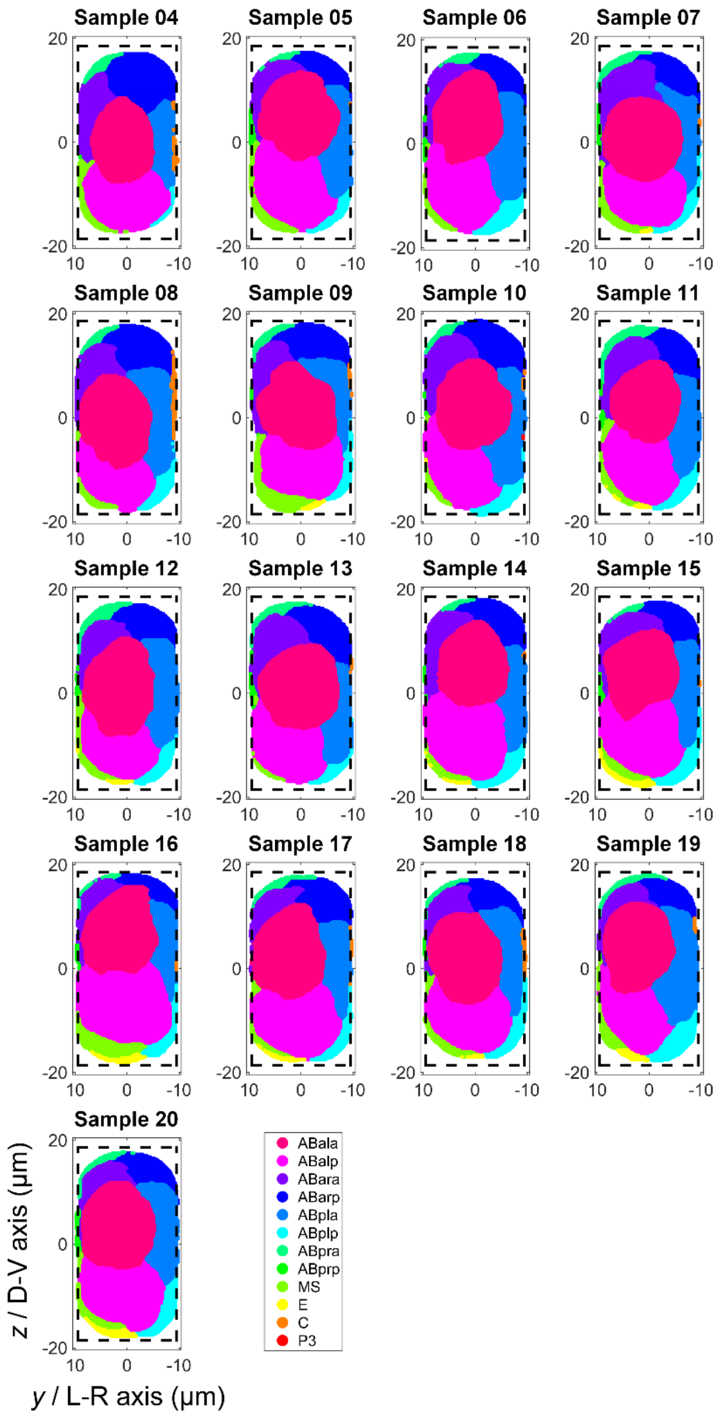

Supplementary Figure 4. 17 standardized embryo samples after spatial normalization (*x* projection/side view). The *y* and *z* axes represent left-right (L-R) and dorsal-ventral (D-V) axes, respectively. Each color represents one specific blast cell as indicated.

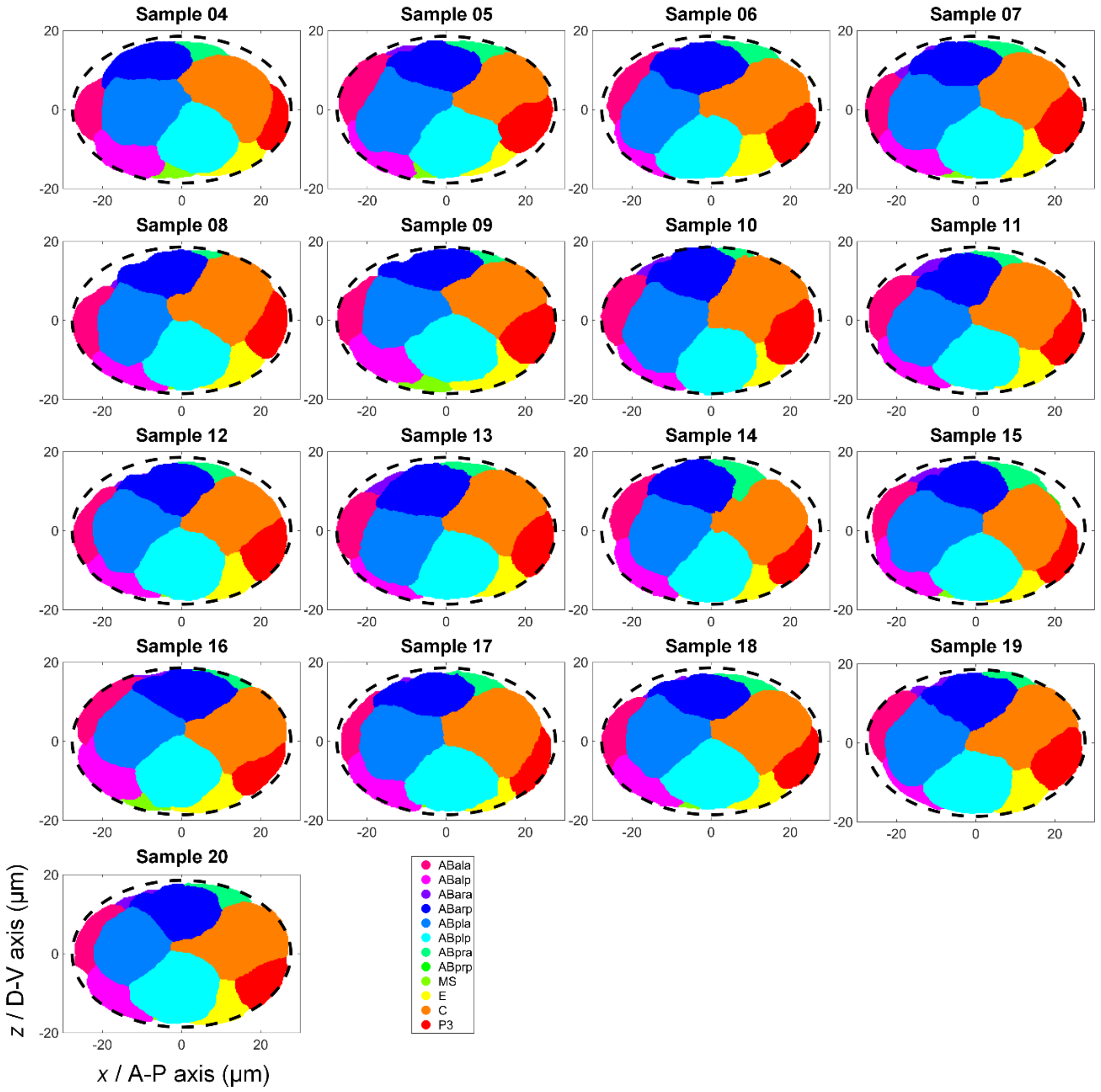

Supplementary Figure 5. 17 standardized embryo samples after spatial normalization (*y* projection/front view). The *x* and *z* axes represent anterior-posterior (A-P) and dorsal-ventral (D-V) axes, respectively. Each color represents one specific blast cell as indicated.

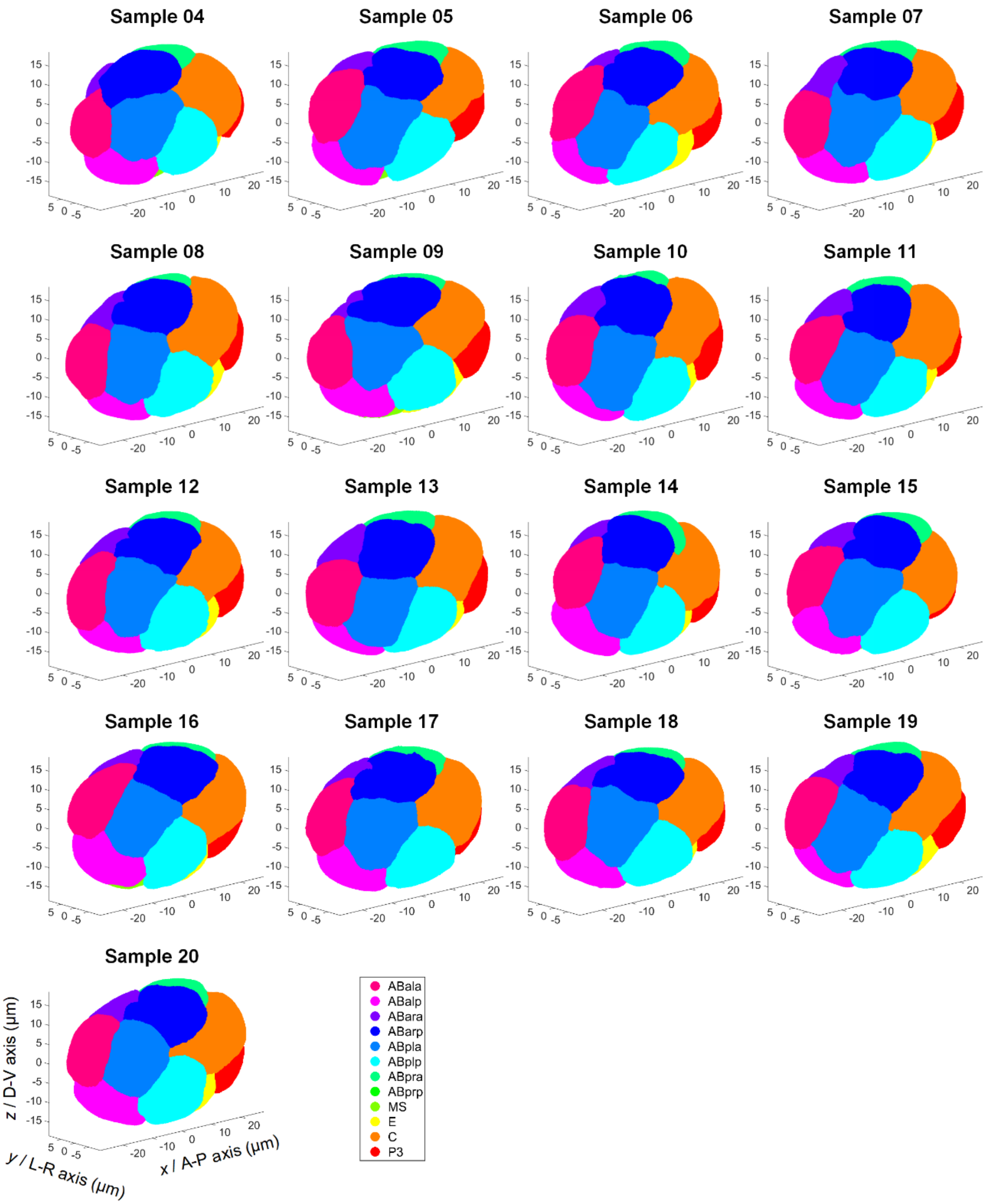

Supplementary Figure 6. Standardized 3D embryos after spatial normalization of 17 membrane-labeled samples. The x, y and z axes represent anterior-posterior (A-P), left-right (L-R) and dorsal-ventral (D-V) axes, respectively. Each color represents one specific blast cell identity as indicated.

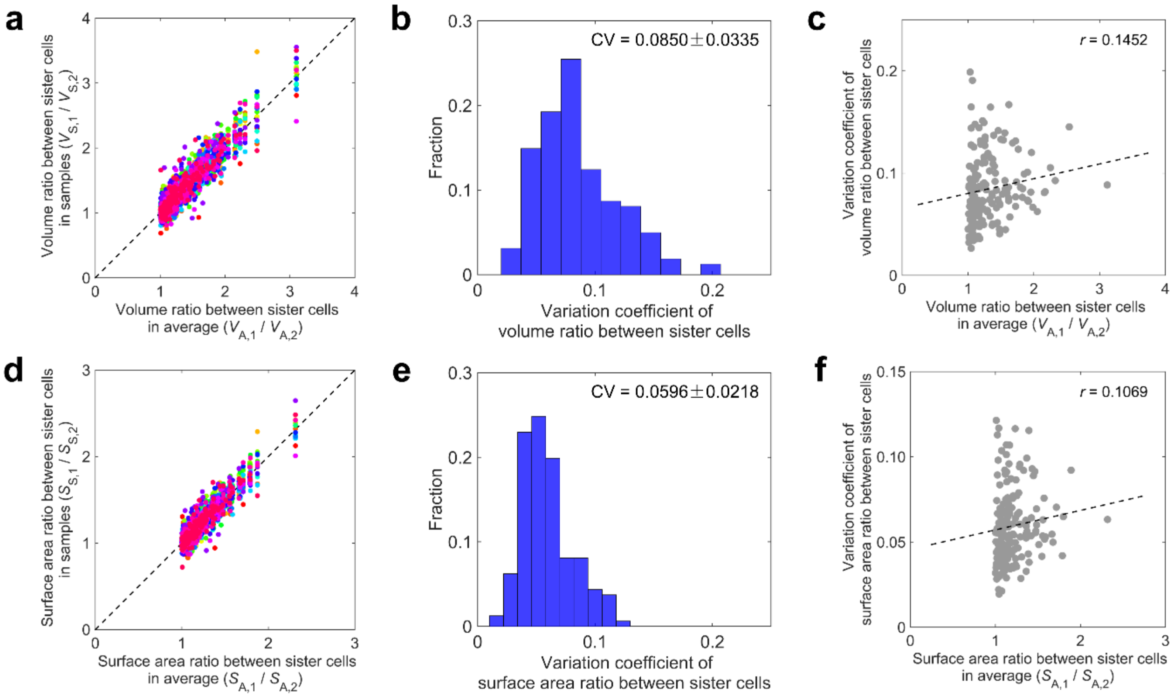

Supplementary Figure 7. Reproducibility and variability of size ratio between sister cells. A total of 161 pairs of sister cells among the 17 wild-type embryos were evaluated. (**a**, **d**) Distribution of ratios for volume (**a**) and surface area (**d**) compared to their averages ($V_{A,1}$ / $V_{A,2} \geq 1$, $S_{A,1}$ / $S_{A,2} \geq 1$). Each color represents an individual embryo. (**b**, **e**) Variation coefficients of each cell pair among the 17 embryos. The average and standard deviation of the variation coefficients are shown on the top right. (**c**, **f**) Correlation between cell size ratios and their variation coefficients among the 17 embryos. The Pearson correlation coefficient r ($r = \frac{\sum x_i y_i - \frac{1}{N}\sum x_i \sum y_i}{\sqrt{[\sum x_i^2 - \frac{1}{N}(\sum x_i)^2][\sum y_i^2 - \frac{1}{N}(\sum y_i)^2]}}$; x_i and y_i, the two groups of variables subjected to test; N, sample size) is shown on the top right, revealing no significant or strong correlation. Source data are provided as a Source Data file.

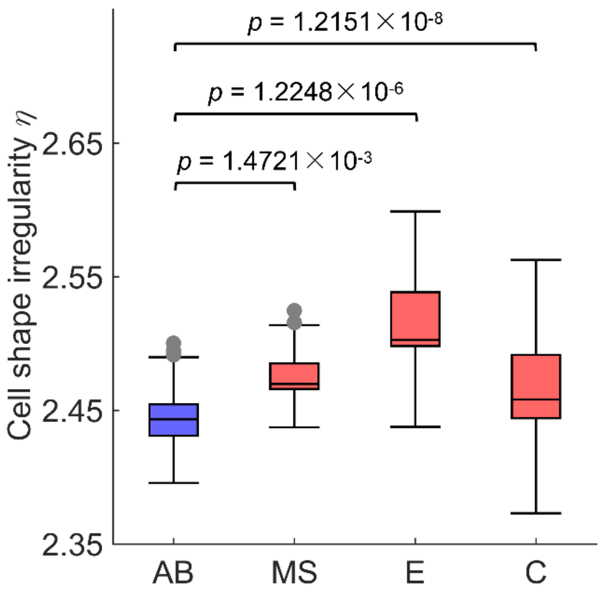

Supplementary Figure 8. Comparison of cell shape irregularity between AB (blue) and P1 (red) lineages, using the last generation of the 322 cells with a complete lifespan. All the sublineages of P1 (i.e., MS, E and C sublineages) are included execpt D, for that its last generation within examination scope consist of only 4 cells (samples), which are not enough for the statistics of box plot. Significance level is derived by one-sided Wilcoxon rank-sum test over n = 128, 16, 8 and 8 independent cells in AB, MS, E and C (sub)lineages respectively, with $p = 1.4721\times10^{-3}$ (AB and MS), 1.2248×10^{-6} (AB and E) and 1.2151×10^{-8} (AB and C), as indicated. The data range between the lower and upper quartiles is illustrated using blue and red boxes for AB and P1 lineages respectively, along with a black line inside indicating the data median. The lower and upper inner fences defined by *Lower Quartile - 1.5× Interquartile Range* and *Upper Quartile + 1.5× Interquartile Range* are represented by two black lines extending from the box. The grey points denote the mild outliers smaller than *Lower Quartile - 1.5× Interquartile Range* or larger than *Upper Quartile + 1.5× Interquartile Range*. Source data are provided as a Source Data file.

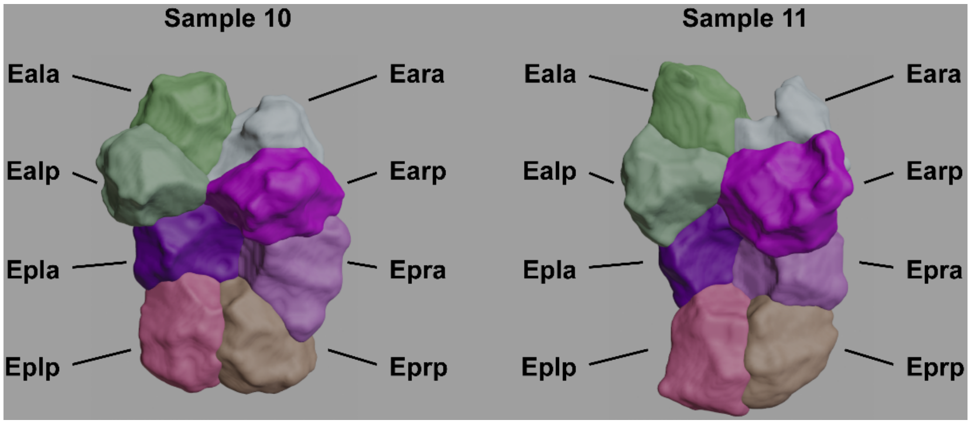

Supplementary Figure 9. Bilaterally symmetric intestine cells at E8 stage. The eight intestine cells present at this stage are illustrated from the view of image shooting perspective from embryo Samples 10 (left) and 11 (right). Each color represents an individual E cell as indicated.

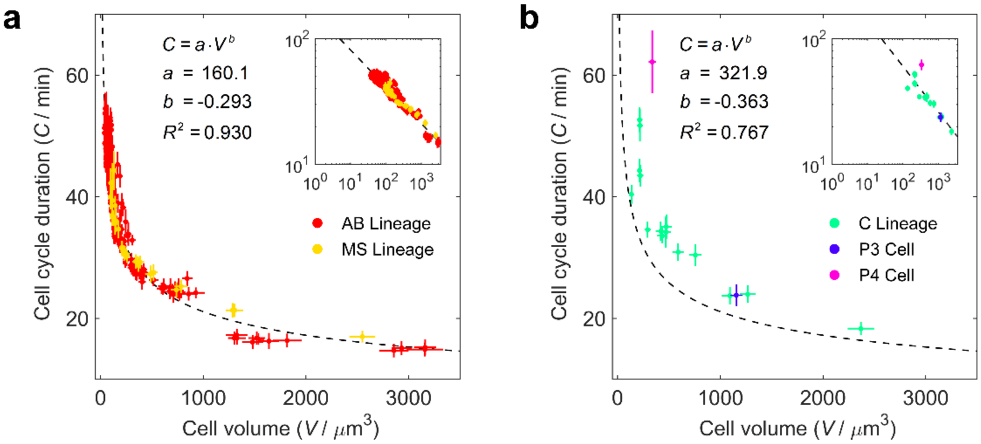

Supplementary Figure 10. Verification of the power law relationship between cell cycle duration and cell volume during development. Cell cycle duration and cell volume of **(a)** AB and MS cells, with a power exponent ≈ -0.293; (**b**) C and P cells, with a smaller power exponent ≈ -0.363. The insets denote the same data with a log-log scale coordinate system. Error bar represents standard deviation (s.d.). Source data are provided as a Source Data file.

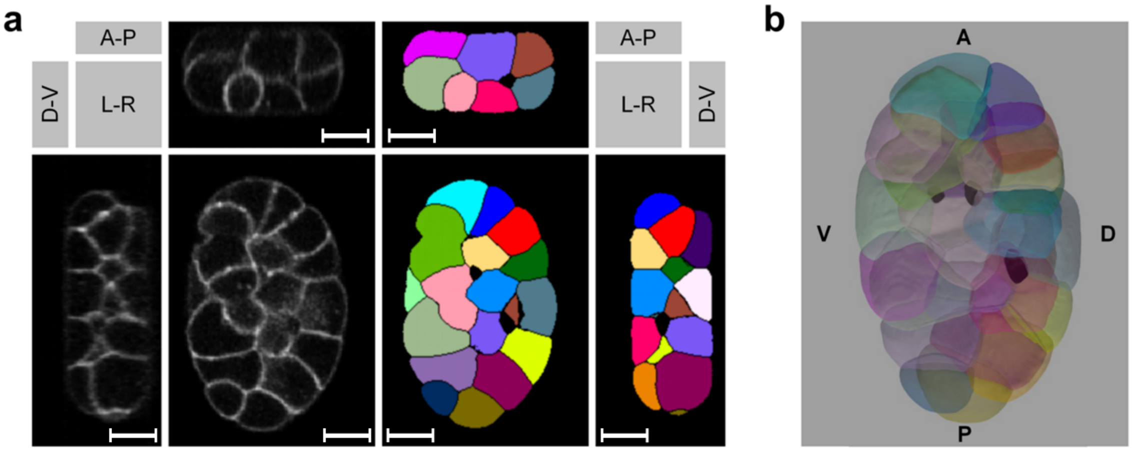

Supplementary Figure 11. Identification of the blastocoel inside *C. elegans* embryo at the 26-cell-stage. Three cavities are identified in embryo Sample 14 at time point 35. (**a**) Cross-sectional microscopy images (left) with segmentations (right). The view directions are as in Supplementary Figure 2. (**b**) Rendered 3D structure. Each color represents one specific blast cell identity. The inner cavities are colored black. D, dorsal; V, ventral; L, left; R, right; A, anterior; P, posterior. Scale bar, 10 µm.

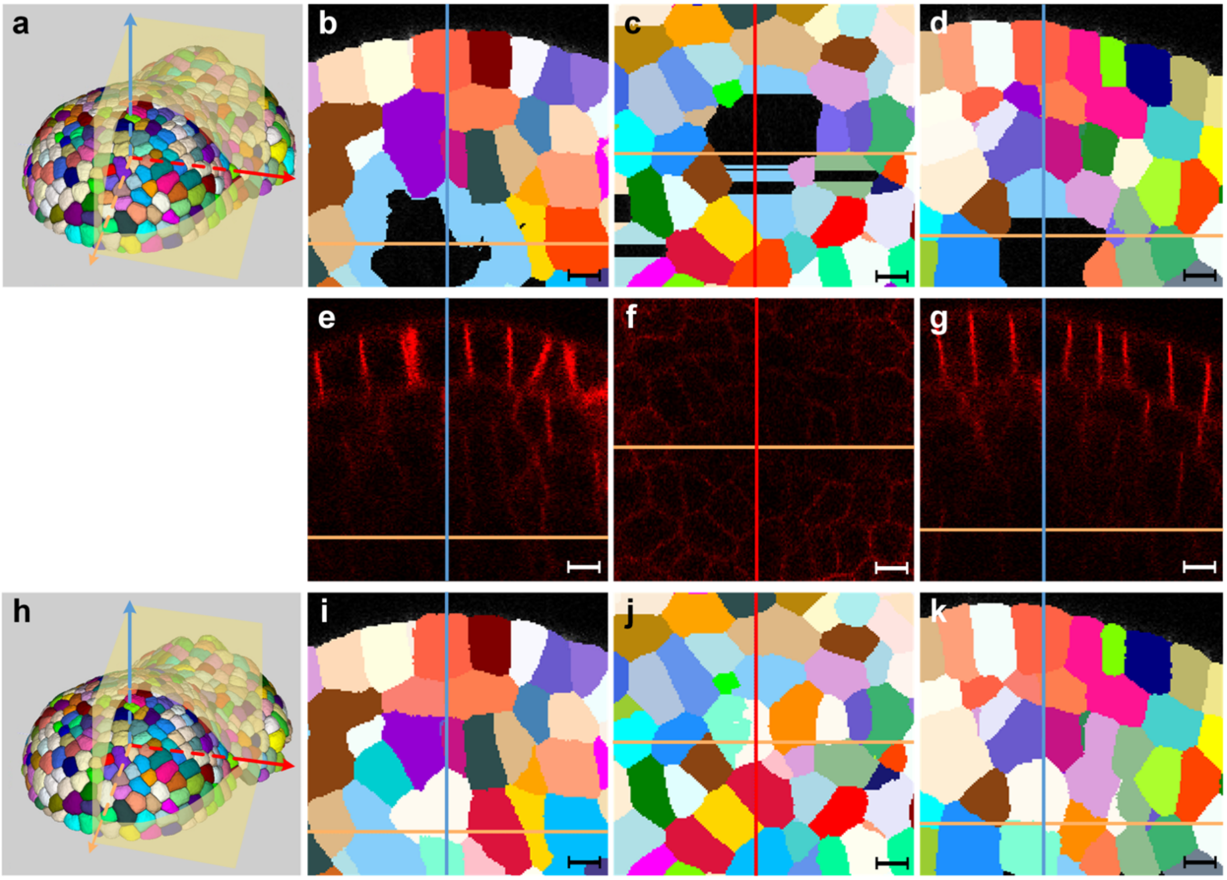

Supplementary Figure 12. Application of CShaper to plant tissue. (**a**, **h**) Segmentations produced by MARS[6] and CShaper, respectively. Three orthogonal sections of (**b**, **c**, **d**) MARS's segmentation, (**e**, **f**, **g**) the raw images and (**i**, **j**, **k**) CShaper's segmentation are listed for comparison. The crossing lines correspond to the axes illustrated in (**a**, **h**). Scale bar, 10 μm.

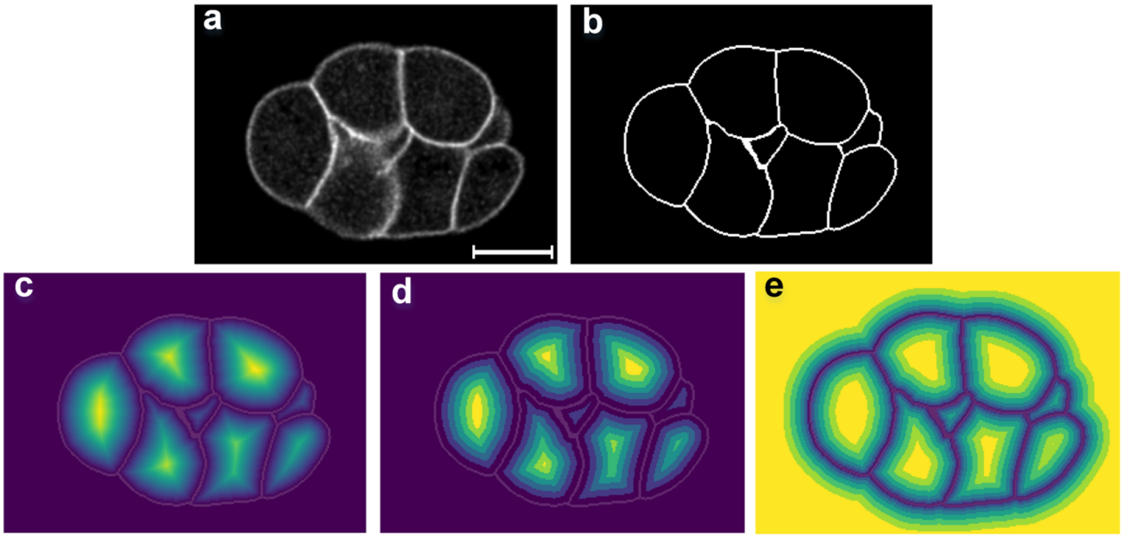

Supplementary Figure 13. The difference of sliced distance maps processed by Wang et al.[4], Bai et al.[7] and CShaper. (**a**) Raw membrane image. (**b**) Membrane annotation. (**c**, **d**, **e**) are the distance maps used by Wang et al., Bai et al. and CShaper, respectively. Translucent binary membrane is overlaid in (**c**), (**d**) and (**e**). In CShaper, the distance map is nonuniformly discretized both inside and outside of the embryo. Scale bar, 10 μm.

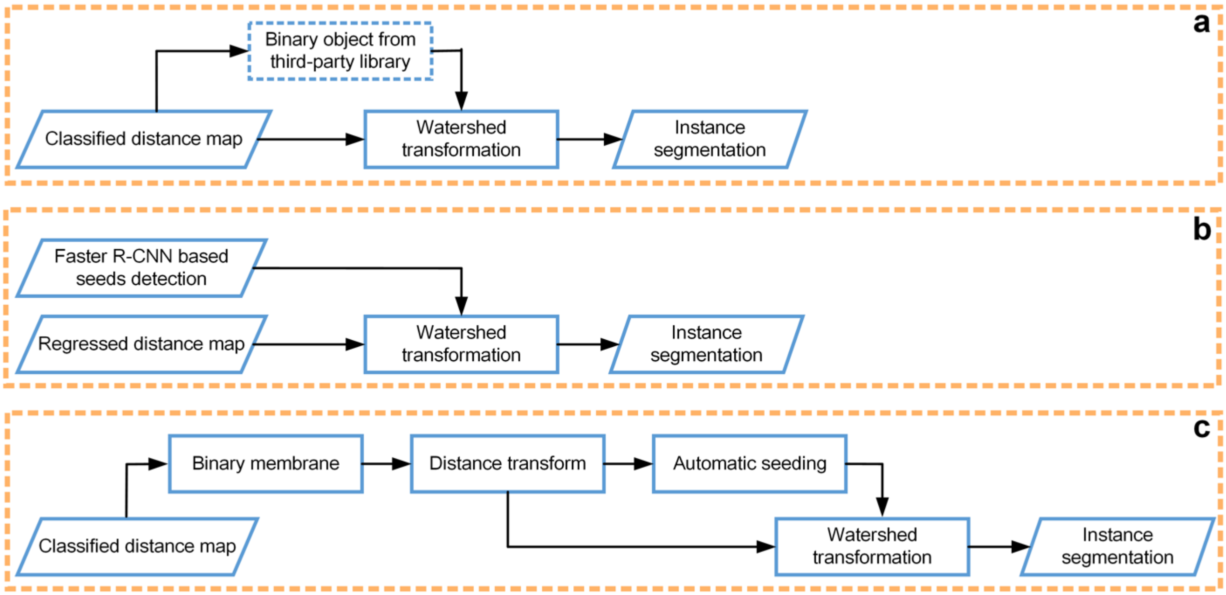

Supplementary Figure 14. Usage of distance map in Wang et al.[4], Bai et al.[7] and CShaper. (**a**) Based on the semantic segmentation, Bai et al. extracted the boundary of each instance based on the distance map. Then each separated region is treated as one object, which can also be regarded as center-seeded watershed segmentation; (**b**) Wang et al. utilized two separated networks to generate continuous distance map and seeds, respectively; (**c**) Differently, after identifying the boundary membrane, CShaper derives seeds with a more reliable seeding procedure to prevent over- and under-segmentation.

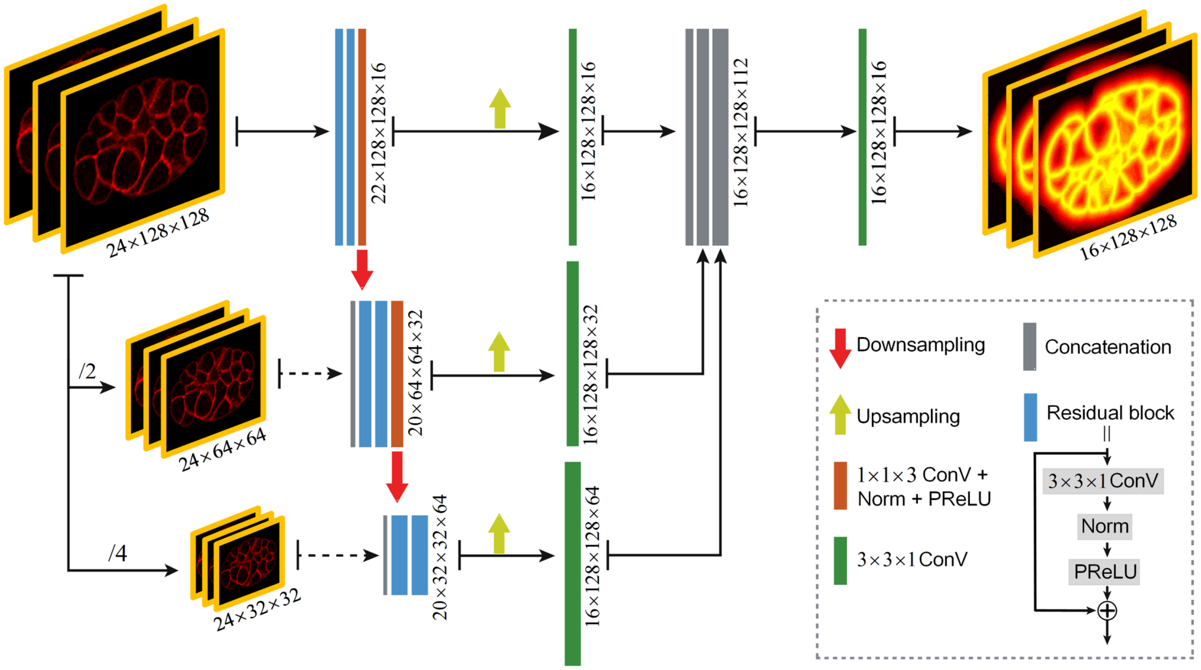

Supplementary Figure 15. The network structure of DMapNet. The number along each layer indicates the dimensions of the output data, which form the input to the next layer. The number of convolution filters at each stage equals to the final output channels.

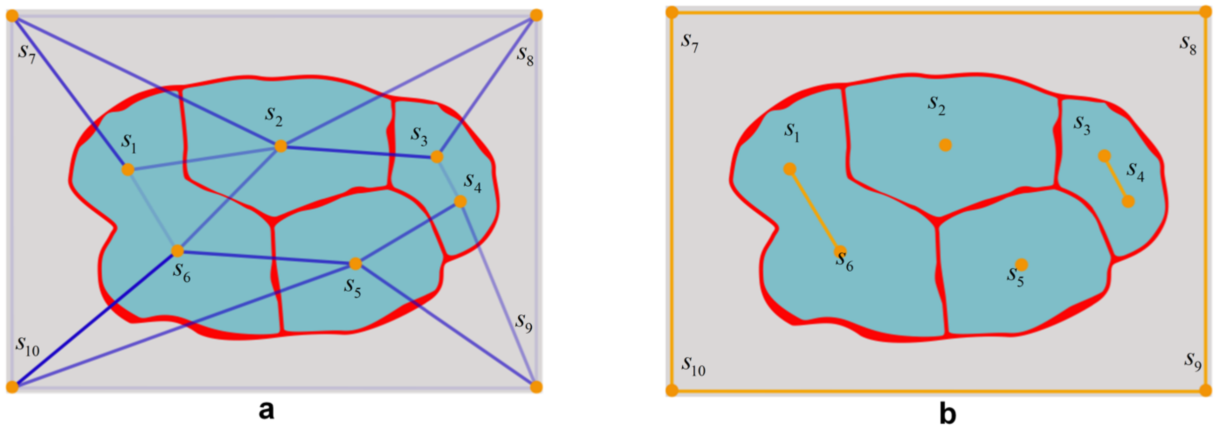

Supplementary Figure 16. Automatic seeding based on the Delaunay triangulation. The red membrane contour corresponds to the *K*-th class predicted by DMapNet. Orange points s_1, ..., s_{10} denotes the local maximum of the distance transformation, which treats the contour as the background. (**a**) While the Delaunay triangulation connects all local maxima s_1, ..., s_{10} as a graph, all edges are weighted by the integrated membrane along the edge (see section Methods). (**b**) Based on this graph, local maxima are adaptively grouped (orange lines) by thresholding the weights.

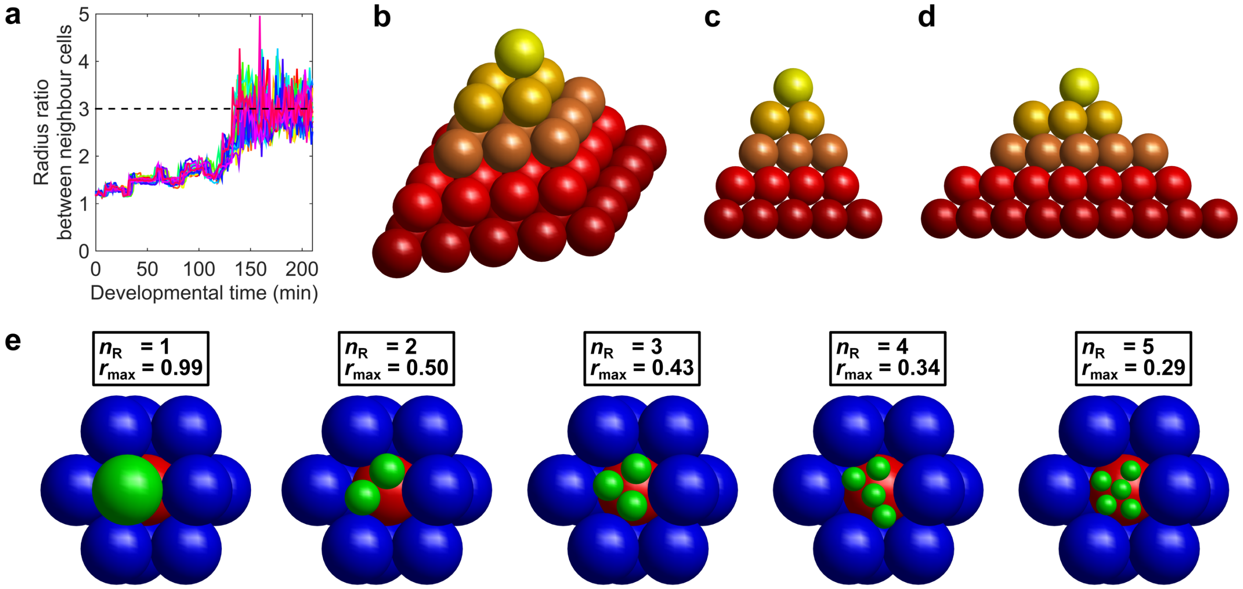

Supplementary Figure 17. Estimation of the sufficient threshold for effective cell-cell contact. (**a**) Radius ratio between neighbour cells over developmental time. The last time point of the 4-cell stage is set as the starting time point. Each color represents one of the 17 embryo Samples (04-20). Hexagonal closely-packed structures from a (**b**) 3D view, (**c**) side view and (**d**) front view. (**e**) A unit cell is replaced by n_R cells with radius r_{max}. The red sphere represents the center cell O_0, and the blue spheres represent the 11 constant neighbors. The green spheres represent the new, smaller cells that replace the original cell, with a maximum radius, r_{max}, obtained from the 10^9 independent trials in the simulation. The r_{max} approaches 1/3 when $n_R = 4$.

Supplementary Table 1. Implementation Details of the Existing Methods

Method	Source Code	Dimension	Output	Post-Process
CellProfiler[1]	https://cellprofiler.org	3D	instance cell	none
RACE[2]	https://bitbucket.org/jstegmaier/race/downloads/	3D	instance cell	none
FusionNet[3]	https://github.com/GunhoChoi/FusionNet-Pytorch	2D	binary membrane	nucleus seeded watershed
SingleCellDetector[4]	https://github.com/opnumten/single_cell_segmentation	2D	instance cell	nucleus seeded watershed
3DUnet[5]	https://github.com/cao13jf/3DUNet.git	3D	binary membrane	nucleus seeded watershed

Supplementary Table 2. Comparison of Object-Level Performance of Methods

Method	Split	Merge	Precision	Recall
3DUNet[5]	**0.21%**	2.44%	86.96%	72.79%
CellProfiler[1]	0.24%	2.02%	93.01%	78.03%
FusionNet[3]	4.32%	1.79%	72.91%	66.66%
RACE[2]	2.95%	6.21%	74.44%	72.39%
SingleCellDetector[4]	2.24%	3.19%	81.95%	80.11%
B-CShaper	7.13%	**0.00%**	84.67%	55.91%
CShaper	0.67%	0.31%	**98.35%**	**97.28%**

Note: All scores are calculated at 0.7 IoU between the ground truth annotation and automatic segmentation. A satisfactory method is expected to not only have relatively small split and merge errors, but also keep high precision and recall scores.

Supplementary Table 3. Cell Loss Ratio under Different Fractions of Lifespan

Fraction of Lifespan	0.1	0.2	0.3	0.4	0.5	0.6	0.7	0.8	0.9	1.0
Cell Loss Ratio	19.96%	12.19%	7.96%	5.77%	4.48%	3.11%	2.32%	1.64%	0.95%	0.44%

Note: Fraction of Lifespan = 0.1 means that a cell is lost within at least 0.1 (included) of its whole lifespan. For example, if the length of a cell's lifespan is 50 time points, it would be lost in at least 5 time points.

Supplementary Table 4. Intrinsic Variability in Embryo Size

Embryo	Embryo Size Relative to Average	Goodness of Fit
Sample 04	0.9073	0.9947
Sample 05	0.9990	0.9941
Sample 06	0.9164	0.9955
Sample 07	0.9818	0.9971
Sample 08	0.9086	0.9956
Sample 09	1.0168	0.9958
Sample 10	1.0675	0.9968
Sample 11	0.9837	0.9944
Sample 12	1.0416	0.9965
Sample 13	1.0615	0.9954
Sample 14	1.0311	0.9959
Sample 15	0.9827	0.9927
Sample 16	1.0690	0.9958
Sample 17	0.9847	0.9971
Sample 18	1.0025	0.9974
Sample 19	1.0073	0.9957
Sample 20	1.0385	0.9952

Note: The goodness of fit is calculated according to $g = 1 - \frac{\Sigma(y_i' - y_i)^2}{\Sigma(y_i - \frac{1}{N}\Sigma y_i)^2}$, where x_i is the average volume of cell i over the 17 embryo samples, y_i is the original volume of cell i in a specific embryo, y_i' is the cell volume predicted based on proportional fitting ($y_i' = x_i \cdot$ Embryo Size Relative to Average).

Supplementary Table 5. Variation Coefficient of Different Cell Size Properties

Cell Size Property	Average ± Standard Deviation	Maximum of 99% Data	Maximum	Minimum
Volume	0.0921 ± 0.0336	0.1999	0.2836	0.0300
Volume Ratio Between Sister Cells	0.0850 ± 0.0335	0.1670	01988	0.0265
Surface Area	0.0644 ± 0.0232	0.1434	0.1883	0.0202
Surface Area Ratio Between Sister Cells	0.0596 ± 0.0218	0.1165	0.1214	0.0195

Supplementary Table 6. Wild-Type Embryo Samples Used in Training and Evaluation

Usage	Embryo	Frame Number / Time Point	Cell Number
Training	Sample 01	Total : 27<br>[30, 31, 32, 33, 34, 35, 36, 37, 50, 51, 52, 53, 54, 55, 56, 57, 64, 67, 70, 73, 76, 79, 82, 85, 88, 91, 94]	Total : 2269<br>[3, 4, 6, 7, 8, 12, 14, 15, 24, 25, 26, 28, 44, 47, 51, 51, 86, 89, 97, 102, 150, 173, 182, 187, 194, 309, 335]
	Sample 02	Total : 27<br>[1, 5, 9, 13, 17, 21, 25, 29, 33, 37, 41, 45, 49, 53, 57, 63, 69, 75, 81, 87, 93, 99, 105, 111, 117, 129, 135]	Total : 1219<br>[12, 13, 14, 14, 14, 15, 15, 15, 26, 26, 28, 28, 28, 42, 43, 44, 49, 51, 51, 53, 85, 86, 87, 92, 94, 97, 97]
Evaluation	Sample 02	Total : 7<br>[24, 34, 44, 54, 64, 74, 84]	Total : 353<br>[13, 24, 28, 47, 51, 87, 103]
	Sample 03	Total : 7<br>[24, 34, 44, 54, 64, 74, 84]	Total : 261<br>[8, 14, 24, 28, 47, 52, 88]
	Sample 04	Total : 7<br>[24, 34, 44, 54, 64, 74, 84]	Total : 470<br>[16, 26, 44, 53, 87, 96, 148]

Supplementary Note 1

In section Results, we compared 3DUnet[5], CellProfiler[1], FusionNet[3], RACE[2], SingleCellDetector[4], B-CShaper and CShaper. Here, we list the various parameters used to implement these methods with our evaluation dataset. In line with the original framework, input and output data flow is adaptively revised if necessary. Additional implementation information about the source codes is available in Supplementary Table 1.

(1) 3DUnet

During the training stage, an input of size $64 \times 64 \times 64$ was randomly cropped from resampled and annotated images of size $205 \times 285 \times 134$. The batch size was set to 4. Adam optimization was used to train the network with an initial learning rate of 1×10^{-4}. Other settings, including the network structure, were kept consistent with the original repository. Considering the limitation of our computation resources, raw images were resized to $144 \times 144 \times 144$, and corresponding output was resized back to the original size during the inference stage. As the output is a binary membrane mask, the nucleus location was used as a seed for the watershed segmentation in the postprocessing stage.

(2) CellProfiler

An advanced pipeline was designed to segment membranes in 3D. Specifically, the membrane channel was processed by *RescaleIntensity*, *GaussianFilter*, *Threshold* and *Watershed3DWithEdt* sequentially. Here, the *Watershed3DWithEdt* is a user-defined tool which can apply watershed transformation to the binary membrane mask with the nucleus centroid as the seed. The CellProfiler project file is publicly available at our code depository.

(3) FusionNet

FusionNet was trained and tested on 2D slices obtained from the volumetric data. Slices were resized to 256×256 in both the training and testing stages. To obtain the volume result, output slices were stacked together after being resized back to the original dimensions. All other parameters were kept as recommended in the FusionNet paper.

(4) RACE

RACE provides a user-friendly GUI tool to segment data. We downloaded the tool and processed *C. elegans* data according to the user's manual. *RACE Seeding* was set to nuclei and *Intensity Parameters* were fixed. We experimentally tuned the *Microscope/Specimen Parameters* via visual inspection of preliminary results. Parameters, such as *Max 3D Cell Volume* and *Max 2D Segmentation Area*, were adaptively changed for the embryo images at different cell stages. All parameter settings for RACE can be found at our code depository.

(5) SingleCellDetector

Based on the framework used for single cell detection, we retrained the network with sliced raw image and annotation pairs. To get similar results as reported in the original paper, only the data loader parameter was changed, while keeping all other parameters as previously reported. However, we did not divide the normalized image intensity by the middle value, which was zero in our case. Segmentation slices were rendered as a 3D volume to obtain our final predicted distance map. We also designed a 3D version of SingleCellDetector, but the corresponding result was of much lower quality than the result of the original 2D SingleCellDetector. This might be due to a lack of sufficient training data. Thus, these results are not reported.

Supplementary Note 2

In CShaper, there are three discriminative situations whereby the nuclei derived from StarryNite and AceTree[8] cannot be successfully paired to a segmented region:

(1) The boundary between two cells (not sisters) is too weak to be extracted by DMapNet. As a result, two cells are segmented as one during the watershed transformation.

(2) Membrane signal is lost at the boundary of the embryo, which leads to the leakage of the background into the embryo.

(3) In CShaper, we determine the accomplishment of a cell division by checking the signal intensity of the line connecting two sister cells' nuclei. However, when the intensity drops at the middle of a cell's lifespan, two cells may be renamed as their mother cell, even though the cell division is completed.

Based on these unsuccessful pairs, we evaluated the segmentation accuracy at the object level in the section Results.

Supplementary Note 3

Given the close-packing structure of equal spheres, the threshold for cell-cell contact area was estimated by solving the problem of how many cells with a radius of 1/3 can occupy the space formed by a neighboring cell with a radius of 1 (Supplementary Figures 17b, c, d, e). To this end, we first generated a hexagonal close-packed structure where a central cell O_0 is surrounded by 12 neighbors $O_{1\sim12}$. By taking O_0 as the origin, we established a spherical coordinate system for these 13-unit cells. Thereafter, we replaced one of the neighboring cells, for example, O_1, with n_R identical cells. To search for the maximum radius r_max that can accommodate n_R cells, we determine n_R cell centers whose inclination and azimuth were sampled from a normal distribution with O_1's center as the mean and $\pi/3$ as the standard deviation. Finally, the radius r_max of the n_R cells was maximized under the condition such that they were exactly in contact with cell O_0 but did not overlap each other. Here, n_R was set as 1-5. Sequentially, we derived the r_max through a trial-and-test method with 10^9 trials based on previous samples.

Noticeably, the threshold value for relative contact area obtained here ($1/48 \approx 2.08\%$) was smaller than the one previously used (6.5%), which was derived from known cell pairs with Notch signaling[9]. However, the contact of the C-ABar cell with Wnt signaling was not previously considered[10]. This contact has a smaller relative contact area such that only 2 of the 17 embryos reach the old threshold value. However, our new assigned value of 1/48 ($\approx 2.08\%$) based on geometrical modeling permits a higher pass rate of 15/17. Considering the outliers of C-ABar and MSappp-ABplpppp contacts, false negatives may be unavoidable, as the actual sensitivity of intercellular signaling is still unknown (see sections Results and Discussion).

Supplementary references

1. McQuin, C. et al. CellProfiler 3.0: next-generation image processing for biology. *PLoS Biol.* **16**, e2005970 (2018).

2. Stegmaier, J. et al. Real-time three-dimensional cell segmentation in large-scale microscopy data of developing embryos. *Dev. Cell* **36**, 225-240 (2016).

3. Quan, T. M., Hildebrand, D. G. & Jeong, W. -K. FusionNet: a deep fully residual convolutional neural network for image segmentation in connectomics. Preprint at https://arxiv.org/abs/1612.05360 (2016).

4. Wang, W. et al. Learn to segment single cells with deep distance estimator and deep cell detector. *Comput. Biol. Med.* **108**, 133-141 (2019).

5. Eschweiler, D. et al. CNN-based preprocessing to optimize watershed-based cell segmentation in 3D confocal microscopy images. *2019 IEEE 16th International Symposium on Biomedical Imaging on 223-227* (ISBI, 2019).

6. Fernandez, R. et al. Imaging plant growth in 4D: robust tissue reconstruction and lineaging at cell resolution. *Nat. Methods* **7**, 547-553 (2010).

7. Bai, M. & Urtasun, R. Deep watershed transform for instance segmentation. *2017 IEEE Conference on Computer Vision and Pattern Recognition on 2858-2866* (CVPR, 2017).

8. Murray, J. I., Bao, Z., Boyle, T. J. & Waterston, R. H. The lineaging of fluorescently-labeled *Caenorhabditis elegans* embryos with StarryNite and AceTree. *Nat. Protoc.* **1**, 1468-1476 (2006).

9. Chen, L. et al. Establishment of signaling interactions with cellular resolution for every cell cycle of embryogenesis. *Genetics* **209**, 37-49 (2018).

10. Walston, T. et al. Multiple Wnt signaling pathways converge to orient the mitotic spindle in early *C. elegans* embryos. *Dev. Cell* **7**, 831-841 (2004).
