# Supplementary material for: Establishment of a morphological atlas of the Caenorhabditis elegans embryo using deep-learning-based 4D segmentation
Source: Nat Commun. 2020 Dec 7;11:6254. doi: 10.1038/s41467-020-19863-x (PMC7721714; doi:10.1038/s41467-020-19863-x)
Supplement: Supplementary file 12 — Description of Additional Supplementary Files [file 41467_2020_19863_MOESM12_ESM.pdf]

Title: Supplementary Movie 1.
Description: Segmentation movie of wild-type *C. elegans* embryo. This movie shows the segmentation results of the wild-type embryo Sample 06. Different cells are labeled with different colors, allowing a time-lapse cellular morphological dynamics during embryogenesis to be visualized at a single-cell resolution.

Title: Supplementary Data 1.
Description: Experimental information of the wild-type embryo samples used in this study. This file details all experimental information of the 49 embryos (Samples 01-49) analysed, including time resolution, resolution along the imaging direction, terminal frame number, terminal cell number, expressed marker as well as the embryos' usage.

Title: Supplementary Data 2.
Description: Timing of conserved developmental landmarks from the 4- to 350-cell stages. This file details the timing of 54 conserved developmental landmarks by average and standard deviation derived from 46 wild-type embryos (Samples 04-49).

Title: Supplementary Data 3.
Description: List of cells present in each embryo. This file details the four cell groups involved in this study as follows, the first four cells, the cells with a complete lifespan recorded, the cells without a complete lifespan recorded but confirmed to appear in at least one time point, and the cells without intentional control.

Title: Supplementary Data 4.
Description: List of cells segmented in each embryo. This file details if the 322 cells with a complete lifespan recorded are successfully segmented throughout their lifespan in each embryo (Samples 04-20).

Title: Supplementary Data 5.
Description: Identified cell-cell contacts. This file details the cell-cell contacts filtered out through different but progressive criteria, including the ones detected with a contact area larger than zero for at least one time point in at least one embryo, the ones with contact area ratio $s/S_i \geq 1/48$ or $s/S_j \geq 1/48$, the ones with contact duration ≥ 2 time points (≈ 3 min), and the ones reproducible in all the 17 wild-type embryos (Samples 04-20).

Title: Supplementary Data 6.
Description: Known cell-cell signaling pairs. This file details the contact information of 10 previously-proposed signalings between specific cells, including contact duration, contact area, maximum contact area, surface area, relative contact area, and their validity and reproducibility in the 17 wild-type embryos (Samples 04-20).

Title: Supplementary Data 7.
Description: Cell shape irregularities. This file details the average cell shape irregularities of the 322 cells with a complete lifespan recorded, which are evaluated by dimensionless surface-to-volume ratio η.

Title: Supplementary Data 8.
Description: Strain information. This file details the information of strains used in this study, including strain name, genotype, phenotype (expressed marker) and insertion site.
