# Supplementary material for: Establishment of a morphological atlas of the Caenorhabditis elegans embryo using deep-learning-based 4D segmentation
Source: Nat Commun. 2020 Dec 7;11:6254. doi: 10.1038/s41467-020-19863-x (PMC7721714; doi:10.1038/s41467-020-19863-x)
Supplement: Supplementary file 13 — Reporting Summary [file 41467_2020_19863_MOESM13_ESM.pdf]

nature research

Corresponding author(s): Chao Tang, Zhongying Zhao, Hong Yan

Last updated by author(s): Sep 9, 2020

Reporting Summary

Nature Research wishes to improve the reproducibility of the work that we publish. This form provides structure for consistency and transparency in reporting. For further information on Nature Research policies, see our Editorial Policies and the Editorial Policy Checklist.

Statistics

For all statistical analyses, confirm that the following items are present in the figure legend, table legend, main text, or Methods section.

n/a	Confirmed	
☐	☒	The exact sample size (*n*) for each experimental group/condition, given as a discrete number and unit of measurement
☐	☒	A statement on whether measurements were taken from distinct samples or whether the same sample was measured repeatedly
☐	☒	The statistical test(s) used AND whether they are one- or two-sided<br>*Only common tests should be described solely by name; describe more complex techniques in the Methods section.*
☒	☐	A description of all covariates tested
☐	☒	A description of any assumptions or corrections, such as tests of normality and adjustment for multiple comparisons
☐	☒	A full description of the statistical parameters including central tendency (e.g. means) or other basic estimates (e.g. regression coefficient) AND variation (e.g. standard deviation) or associated estimates of uncertainty (e.g. confidence intervals)
☐	☒	For null hypothesis testing, the test statistic (e.g. *F*, *t*, *r*) with confidence intervals, effect sizes, degrees of freedom and *P* value noted<br>*Give P values as exact values whenever suitable.*
☒	☐	For Bayesian analysis, information on the choice of priors and Markov chain Monte Carlo settings
☒	☐	For hierarchical and complex designs, identification of the appropriate level for tests and full reporting of outcomes
☐	☒	Estimates of effect sizes (e.g. Cohen's *d*, Pearson's *r*), indicating how they were calculated

Our web collection on statistics for biologists contains articles on many of the points above.

Software and code

Policy information about availability of computer code

Data collection	Leica TCS SP5 (https://www.leica-microsystems.com/pt/produtos/microscopios-confocais/detalhes/product/show/Products/leica-tcs-sp5/); Version used in this study: LAS AF 2.7.2.9586;<br>Leica TCS SP8 (https://www.leica-microsystems.com/pt/produtos/microscopios-confocais/detalhes/product/show/Products/leica-tcs-sp8/); Version used in this study: LAS X 2.0.1.14392;<br>In this study, Leica TCS SP5/SP8 were used to collect all the raw fluorescent images of C. elegans embryos, including nucleus and membrane.<br><br>Huygens (https://svi.nl/HomePage); Version used in this study: 18.04;<br>Huygens is a platform including multiple imaging processing packages for both 2D and 3D microscopies.<br>In this study, Huygens was used for the restoration of fluorescent images through deconvolution procedure.
Data analysis	Matlab (https://www.mathworks.com/products/matlab.html); Version used in this study: R2018b;<br>Matlab is a commercial tool with many tool boxes, including image processing, control system simulation, etc.<br>In this study, Matlab was used to preprocess raw images, evaluate segmentation performance, analyze the output and generate the standardized morphological dataset.<br><br>ITK-SNAP (http://www.itksnap.org/pmwiki/pmwiki.php); Version used in this study: 3.6.0;<br>ITK-SNAP is a free application used to segment 3D medical images. It has user-friendly GUI and functions to facilitate semi-automatic segmentations.<br>In this study, after using ITK-SNAP to import the initial 3D embryo segmentations from the 3DMMS, segmentations with manual correction of errors were used as the training set for evaluation of datasets.<br><br>StarryNite (http://starrynite.sourceforge.net); Version used in this study: [DOI: 10.1186/1471-2105-15-217];

StarryNite is a free software package for identifying and tracing fluorescently-labeled cell nuclei in C. elegans embryos.

AceTree (http://waterston.gs.washington.edu/AceTree.html); Version used in this study: [DOI: 10.1186/s12859-018-2127-0];
AceTree is a cell lineage editor through which one can manually fix the errors in nucleus identification and tracing by StarryNite.

TensorFlow (https://www.tensorflow.org); Version used in this study: Tensorflow-gpu 2.2.0;
TensorFlow is an open source platform for machine learning. The deep learning model DMapNet used in CShaper was constructed with TensorFlow libraries;

Custom codes were used to segment the C. elegans embryos. Source code is publicly available in the Github repository [https://github.com/cao13jf/CShaper.git]

For manuscripts utilizing custom algorithms or software that are central to the research but not yet described in published literature, software must be made available to editors and reviewers. We strongly encourage code deposition in a community repository (e.g. GitHub). See the Nature Research guidelines for submitting code & software for further information.

Data

Policy information about availability of data

All manuscripts must include a data availability statement. This statement should provide the following information, where applicable:

- Accession codes, unique identifiers, or web links for publicly available datasets
- A list of figures that have associated raw data
- A description of any restrictions on data availability

The datasets generated and analyzed in this study are available at the figshare repository [https://doi.org/10.6084/m9.figshare.12839315.v4]. The raw confocal microscopies before deconvolution are available upon reasonable request.

Field-specific reporting

Please select the one below that is the best fit for your research. If you are not sure, read the appropriate sections before making your selection.

☒ Life sciences ☐ Behavioural & social sciences ☐ Ecological, evolutionary & environmental sciences

For a reference copy of the document with all sections, see nature.com/documents/nr-reporting-summary-flat.pdf

Life sciences study design

All studies must disclose on these points even when the disclosure is negative.

Sample size

No specific statistical method was used to determine the embryo sample sizes in both parts of segmentation algorithm and imaging experiment.

For the segmentation algorithm, there are 54 volumes (from 2 samples) and 21 volumes (from 3 samples) used for training and evaluating the network, respectively. The number and group of samples are chosen to help the deep learning model learn effectively as well as validate its generalization on different imaged samples sufficiently. The trained network can segment embryos with cell loss ratio below 5% up to the 200-cell stage, reaching our expected performance. These samples need to be segmented semi-automatically, which is labor-intensive and time-consuming, and thus limits the sample size we can afford.

For the imaging experiment, membrane segmentation on 17 embryos and nucleus tracing on a total of 46 (17+29) embryos are performed and used for further analysis (Supplementary Data 1). The embryos with membrane information are increased until that all the 322 cells with a complete lifespan were successfully segmented without any frames lost in at least three embryos. Since a recently published paper systematically analyzing C. elegans cell/nucleus position used a sample size of 28 [DOI: 10.1016/j.celrep.2018.12.052], we collected more embryos (29 with only nucleus marker and 46 in total) to achieve a high performance in spatial and temporal normalization.

Data exclusions

A total of 52 embryos are collected and imaged. Two of them fail to be segmented for all the cells in at least one frame, and excluded in the analysis. The other 50 embryos are all used in this study (Supplementary Data 1).

Replication

All attempts at replication are confirmed to be successful.

The performance of CShaper is evaluated on 3 different embryo samples with total 21 images, which shows high accuracy and reproducibility regarding the Wilconxon rank-sum test, low variation and low cell loss ratio (Figures 2a, b, c, d, e).

Derivation of effective contact (Figure 3f; relative contact area $\geq 1/48$, continuous contact duration ≥ 3 min) and illustration on embryo morphology (Figure 3c and Supplementary Figures S4, S5, S6) are performed using all the 17 embryo samples.
Analysis on cell size (Figures 3d, e; goodness of fit > 0.99, CV < 0.2), cell size ratio (Supplementary Figures 7a, b, c, d, e, f) and power law relationship between cell cycle duration and cell volume (Supplementary Figures 10a, b) is performed using all the 17 embryo samples.
Natural variability of shape dynamics including orientation bias, separation time and contact state (Figure 5a), is illustrated with at least 2 replicates, using 6 embryo samples in total, for that they can together represent and summarize the variable dynamics well.
Natural variability of contact state, especially for the connecting state, is illustrated using all the 17 embryo samples (Figure 5b).

Randomization

6 embryos were chosen to summarize the variable cell shape dynamics, including orientation bias, separation time and contact state (Figure

5a). Because this figure is to show the natural variability, there's no need to show all the embryo data, and the selected 6 embryo samples can represent and illustrate the observations well (with at least 2 replicates for each described phenomenon). For the other experiments and analyses, the representative samples were chosen randomly without subjective influence.

Blinding: The blinding method is not used in this study, which only reports the results from imaged embryos without the comparison of treated and untreated groups. There is only one kind of biological system investigated, the C. elegans wild-type embryos, and all the comparative analysis is performed on the objects with completely same methods (e.g. comparison of irregularity score η between AB128 and E8 cells, Figure 4b).

Reporting for specific materials, systems and methods

We require information from authors about some types of materials, experimental systems and methods used in many studies. Here, indicate whether each material, system or method listed is relevant to your study. If you are not sure if a list item applies to your research, read the appropriate section before selecting a response.

Materials & experimental systems

n/a	Involved in the study	
x		Antibodies
x		Eukaryotic cell lines
x		Palaeontology and archaeology
	x	Animals and other organisms
x		Human research participants
x		Clinical data
x		Dual use research of concern

Methods

n/a	Involved in the study	
x		ChIP-seq
x		Flow cytometry
x		MRI-based neuroimaging

Animals and other organisms

Policy information about studies involving animals; ARRIVE guidelines recommended for reporting animal research

Laboratory animals	All C. elegans transgenic strains were built in unc-119(tm4063) background, and the transgenes were maintained as a homozygote in hermaphrodite for imaging of either embryo, larvae, or adult.
Wild animals	Wild animal is not involved in this study.
Field-collected samples	Field-collected sample is not involved in this study.
Ethics oversight	Since invertebrates are involved in the study, ethical approval is not required.

Note that full information on the approval of the study protocol must also be provided in the manuscript.
